# Metacognitive Filtering and Cognitive Offloading in AI-Assisted L2 Writing: A PRISMA Guided Process-Tracing Synthesis

**DOI:** 10.3390/bs16071229

**Published:** 2026-07-20

**Authors:** Latifah Hamdan Alghamdi, Talal Musaed Alghizzi

**Affiliations:** 1 English Language Center, College of Languages and Translation, King Khalid University, Abha 61421, Saudi Arabia; 2 College of Languages and Translation, Imam Mohammad Ibn Saud Islamic University (IMSIU), Riyadh 11564, Saudi Arabia

**Keywords:** artificial intelligence, L2 writing, automated writing evaluation, process tracing, construct validity, feedback uptake, cognitive regulation, authorship, learning analytics, generative AI

## Abstract

This study conducted a PRISMA-guided systematic review of 33 empirical studies examining learner interactions with AI-mediated feedback in L2 writing between 2010 and 2025. Evidence was synthesized from process-tracing methods, including keystroke logging, eye-tracking, screen capture, interaction logs, and draft-history analytics. The findings revealed two recurring interaction profiles. Higher-regulation learners typically engaged in selective uptake, recursive evaluation, and extended processing of AI feedback, whereas lower-regulation learners more frequently indicate rapid acceptance and reduced evaluative engagement. Across studies, micro-level feedback was associated with shorter revision latencies and burst-editing patterns, whereas macro-level feedback was associated with longer processing times and repeated revision cycles. The review also identified substantial methodological variability in trace precision, AI transparency, multimodal triangulation, and reproducibility practices. Overall, the evidence suggests that the effectiveness of AI-mediated feedback depends not only on the quality of the feedback provided but also on how learners regulate and engage with it during revision. Greater methodological transparency and more rigorous process-tracing designs are needed to strengthen future research on AI-supported writing development.

## 1. Introduction

The integration of artificial intelligence (AI) into second language (L2) writing instruction has fundamentally reconfigured the ecology of feedback, revision, and authorship. Automated Writing Evaluation (AWE) systems and large language models (LLMs) now provide real-time linguistic corrections, discourse-level suggestions, and even generative content, reshaping how learners compose, revise, and conceptualize textual ownership (Barrot, 2023; Li et al., 2015; Ranalli, 2018). While early AWE research focused primarily on accuracy gains and error reduction, contemporary AI-mediated environments increasingly influence higher-order dimensions of writing, including cohesion, argumentation, and rhetorical structuring (V. Zhang & Hyland, 2023). Recent evidence suggests that the effectiveness of generative AI feedback depends not only on the quality of the feedback provided but also on learners’ engagement with, evaluation of, and incorporation of AI-generated suggestions during revision. Studies have reported substantial variation in feedback uptake, revision behavior, and writing outcomes, highlighting the importance of learner engagement and feedback literacy in AI-supported writing environments (Ding et al., 2026; Ma et al., 2026).

Despite demonstrated performance improvements, concerns have emerged regarding the cognitive, epistemic, and validity implications of AI-assisted composing. Generative systems may alter revision behaviors, attenuate metacognitive monitoring, or redistribute decision-making authority between the human writer and algorithmic agent (Godwin-Jones, 2024; Kasneci et al., 2023). From an argument-based validity perspective (Kane, 2013), such shifts raise critical questions about construct representation: if AI substantively contributes to textual production, do assessment scores continue to reflect the intended construct of individual L2 writing ability? For example, AI-generated feedback may reshape the writing construct when learners rely on automated suggestions to reformulate sentences, reorganize arguments, improve cohesion, or generate content that would otherwise require independent linguistic and rhetorical decision-making (Godwin-Jones, 2024; Kasneci et al., 2023). In such cases, observed improvements in the final text may reflect not only the learner’s writing ability but also the AI system’s cognitive contribution.

Recent research suggests that AI-mediated feedback can influence revision strategies, feedback uptake patterns, and the distribution of cognitive effort during writing, thereby altering how learners engage with the composing process (Barrot, 2023; V. Zhang & Hyland, 2023). A learner who critically evaluates, modifies, and selectively incorporates AI suggestions may demonstrate substantially different writing processes from a learner who accepts recommendations with minimal scrutiny, even when the resulting texts appear similar (Ranalli, 2018; Koltovskaia, 2020). These distinctions are particularly important for construct validity because they raise questions about whether assessment outcomes represent individual writing competence, human–AI co-constructed performance, or a combination of both, thereby challenging traditional assumptions about authorship and score interpretation (Kane, 2013; Kasneci et al., 2023; Alghamdi & Alghizzi, 2025).

Process-tracing methodologies such as keystroke logging, eye-tracking, screen capture, and timestamped revision analytics offer a mechanism-sensitive lens for addressing these concerns. Unlike product-based analyses, process tracing captures temporal dynamics, revision trajectories, and feedback uptake behaviors, enabling fine-grained modeling of cognitive engagement (Chan, 2017; Révész et al., 2016). Recent studies demonstrate that writing-quality outcomes often mask heterogeneous interaction patterns, with high-regulation writers engaging in recursive evaluation cycles, whereas low-regulation writers exhibit rapid acceptance or cognitive delegation (Barkaoui, 2014). However, the methodological landscape remains fragmented. Reporting transparency regarding AI versions, parameter settings, and tracing infrastructure is inconsistent, limiting reproducibility in rapidly evolving system environments (Kasneci et al., 2023; Godwin-Jones, 2024). Accordingly, a systematic, process-oriented synthesis is needed to consolidate empirical patterns, evaluate methodological robustness, and clarify the validity implications of AI-mediated feedback in L2 writing.

In this light, AI-mediated feedback can be viewed as a process-oriented form of writing support whose effectiveness depends on how learners engage with and regulate feedback during revision (Li et al., 2015; Ranalli, 2018; Barrot, 2023). These interactions lie on a continuum between metacognitive filtering, whereby learners critically evaluate and selectively incorporate AI suggestions, and cognitive offloading, whereby aspects of revision and decision making are delegated to the AI system (Godwin-Jones, 2024; Kasneci et al., 2023). Because similar text outcomes may result from markedly different levels of learner contribution, these processes have important implications for authorship and construct validity.

### 1.1. Context and Significance

Despite growing evidence that AI-mediated feedback can improve writing outcomes, considerably less is known about how learners engage with AI suggestions during the writing process. While existing research has documented gains in accuracy, cohesion, and learner perceptions, the cognitive and behavioral mechanisms underlying feedback uptake remain insufficiently understood. Understanding these mechanisms is essential because writing development depends not only on textual outcomes but also on how feedback is interpreted, evaluated, and incorporated during revision.

### 1.2. Problem Statement and Conceptual Gap

To date, the literature on AI-assisted writing feedback remains heavily product-oriented. Studies typically examine post-test improvements, reductions in error, gains in cohesion or accuracy, or learner satisfaction. What remains underexplored are the microgenetic, moment-by-moment behaviors that unfold as learners interact with AI systems:

- How do learners navigate multiple suggestions?
- When do they hesitate, revise, or ignore feedback?
- What cognitive or metacognitive states are implicated in their revision pathways?
- Which AI features shape uptake, trust, or over-reliance?

Real-time process-tracing methods such as keystroke logging, draft-history analytics, eye-tracking, screen recordings, process mining, and timestamped interaction logs offer unprecedented visibility into these hidden mechanisms. These approaches can reveal temporal patterns, indicators of cognitive load, and decision-making trajectories that cannot be captured by text-only products. However, evidence in this domain is fragmented across disciplines, employs inconsistent definitions, and relies on diverse analytic pipelines, making it difficult to compare findings or build cumulative knowledge. Despite the methodological sophistication of tracing technologies, no existing systematic review has synthesized real-time evidence on how learners engage with AI-assisted feedback. Existing reviews focus on correctness, writing gains, or learner perceptions, leaving a fundamental gap in our understanding of how writing unfolds during AI interaction. More fundamentally, the field remains dominated by outcome-oriented assumptions that treat AI as a performance-enhancing tool rather than a process-mediating cognitive agent. The present review, therefore, shifts analytical attention from writing outcomes to the temporal, metacognitive, and behavioral mechanisms through which AI-mediated revision unfolds. To address this gap, this review synthesizes process-tracing evidence from keystroke logging, eye tracking, screen capture, interaction logs, and time-stamped revisions to explain how learners engage with, evaluate, and incorporate AI feedback during L2 writing.

### 1.3. Purpose and Contribution of the Study

This study addresses these gaps by conducting the first PRISMA-guided systematic review and methodological synthesis of real-time process-tracing research on AI-assisted text feedback. By integrating evidence from multiple tracing paradigms, this review aims to:

1. Identify and characterize the temporal patterns of learner–AI interaction during feedback reception and revision.
2. Explain the cognitive, metacognitive, and behavioral processes inferred from trace indicators such as pauses, fixations, bursts, and revision sequences.
3. Critically evaluate methodological practices, analytic pipelines, and reporting standards in existing studies to propose a coherent framework for future empirical research.

Through this dual empirical–methodological contribution, the review advances the field in three ways. First, it shifts attention from text outcomes to revision pathways, providing a nuanced account of writing-as-process in AI-supported environments. Second, it synthesizes cognitive and behavioral evidence to clarify mechanisms of feedback uptake, trust calibration, and potential cognitive offloading. Third, it establishes a process-tracing methodological framework that can guide rigorous future studies and support replicability in an emerging but methodologically fragmented research area. In doing so, the review reframes AI-assisted writing not primarily as a question of effectiveness, satisfaction, or product improvement, but as a question of construct representation, cognitive redistribution, and revision ecology. This repositioning enables a mechanism-sensitive understanding of how human–AI interaction reshapes writing behavior over time. As a result, the review aims to address these questions:

RQ1. What temporal interaction patterns emerge when learners engage with AI-assisted text feedback, as evidenced through real-time process-tracing methods such as keystroke logging, eye-tracking, draft-history analytics, and interaction logs?
RQ2. What cognitive, metacognitive, and behavioral processes can be inferred from real-time trace indicators during learners’ uptake, evaluation, and modification of AI-generated feedback across different tasks, proficiency levels, and AI tool types?
RQ3. How do current real-time process-tracing studies on AI-assisted feedback differ in methodological design, data reporting, analytic pipelines, and validity practices, and what framework can be proposed to strengthen future research in this domain?

## 2. Methodology

### 2.1. Review Design and Rationale

This study employed a PRISMA 2020-guided systematic review (Page et al., 2021) to synthesize empirical research on real-time process tracing in AI-assisted text feedback. Given the methodological heterogeneity across applied linguistics, educational technology, human–computer interaction, and learning analytics, a structured and transparent review design was required. The review combined systematic retrieval and screening procedures with a structured data extraction framework and a methodological appraisal layer specific to process-tracing research. The final corpus comprised 33 empirical studies that met all inclusion criteria.

### 2.2. Protocol and Reporting Standards

**Figure 1:**
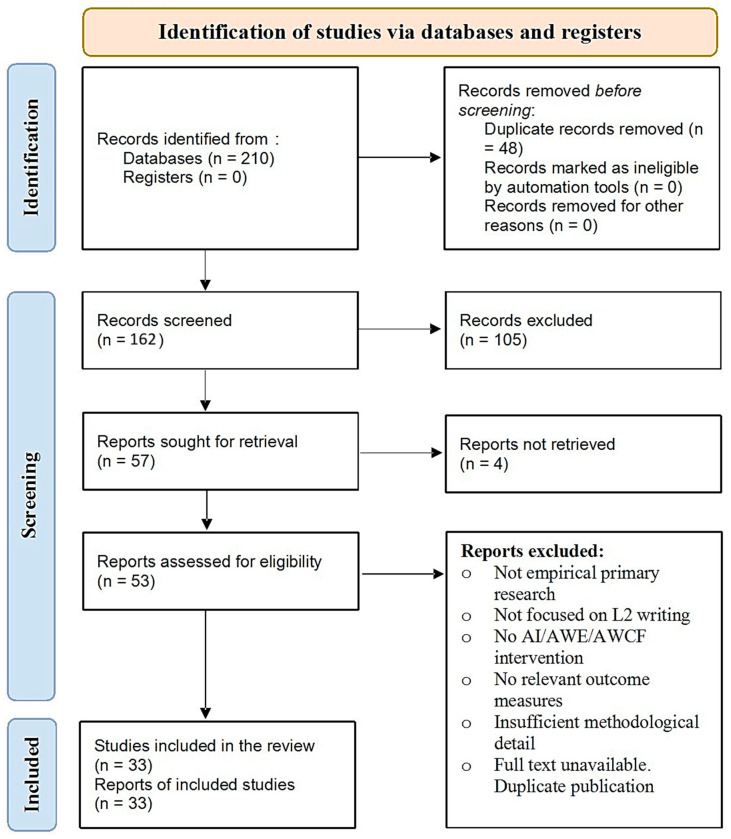
PRISMA 2020 flow diagram illustrating study identification, duplicate removal, screening, eligibility assessment, exclusion procedures, and final study inclusion.

This systematic review was conducted and reported in accordance with the Preferred Reporting Items for Systematic Reviews and Meta-Analyses (PRISMA 2020) Statement. Screening, eligibility assessment, data extraction, methodological appraisal, and synthesis procedures were aligned with PRISMA 2020 reporting standards to ensure methodological transparency and reproducibility. The completed PRISMA 2020 Checklist, reproduced from the PRISMA 2020 Statement (Page et al., 2021), is provided as Supplementary File S1, and the PRISMA 2020 flow diagram is presented in Figure 1.

A structured review protocol was developed prior to data collection and specified the review questions, eligibility criteria, databases, search strategy, screening procedures, coding framework, methodological appraisal criteria, and synthesis plan. However, the protocol was not formally preregistered in PROSPERO or the Open Science Framework (OSF), as the review did not involve a clinical or health-intervention outcome.

### 2.3. Information Sources and Search Strategy

Systematic searches were conducted in Web of Science Core Collection, Scopus, ERIC, and ProQuest. To supplement the indexed literature and reduce publication bias, additional searches were conducted across relevant conference proceedings (e.g., LAK, CHI, AIED), institutional repositories, and doctoral dissertation databases. The search window covered January 2010 to December 2025, with the final search conducted in January 2026. Search strings combined three conceptual clusters: AI-mediated feedback (e.g., automated writing evaluation, AWE, GPT, large language model), process-tracing methodologies (e.g., keystroke logging, eye-tracking, screen capture, process mining, revision logs), and writing contexts (e.g., L2 writing, ESL, EFL, academic writing, revision behavior). Boolean operators were adapted to each database. Database-specific search strategies are provided in Appendix A and reproduced in Supplementary File S2.

#### Search Strings

Search strategies were constructed using a structured, cluster-based approach to ensure conceptual precision and comprehensive retrieval. Three core keyword clusters were combined: (1) AI-mediated feedback (e.g., “automated writing evaluation,” AWE, AWCF, “AI feedback,” GPT, “large language model,” “neural feedback”); (2) process-tracing methodologies (e.g., “keystroke logging,” “eye-tracking,” “screen capture,” “process mining,” “revision logs,” “timestamped analytics”); and (3) L2 writing contexts (e.g., “L2 writing,” ESL, EFL, “revision behaviors,” “academic writing”). Table 1 shows search strategies that combined three keyword clusters.

### 2.4. Eligibility Criteria

Studies were included if they involved human learners producing written text, incorporated AI-mediated feedback (including AWE systems, LLM-based tools, grammar or clarity tools, or hybrid AI–human feedback environments), and employed at least one real-time or quasi-real-time process-tracing method such as keystroke logging, eye-tracking, screen capture, or timestamped interaction logs. Eligible studies were required to report extractable temporal, behavioral, or cognitive indicators and to be empirical in design. Publications had to appear in peer-reviewed venues or reputable grey literature and be written in English. Studies were excluded if they reported only post-test writing outcomes without process-level evidence, used AI solely for text generation without feedback interaction, lacked sufficient methodological detail to permit extraction of trace indicators, or were conceptual or editorial in nature.

#### Data Extraction Framework

A structured extraction matrix was developed to capture study characteristics, process-tracing indicators, and methodological features. Extracted information included bibliographic metadata, participant characteristics, writing task type, AI tool description, and process-tracing methodology. Temporal indicators such as pauses, latency, and revision duration were recorded alongside cognitive and behavioral indicators inferred from trace data. Analytical techniques, validity and reliability reporting, ethical considerations, and author-reported limitations were also documented (see Table 2). All data were entered into a master extraction matrix. A subset of 25% of studies was independently double-coded, yielding inter-coder agreement ranging from κ = 0.84 to 0.91 across coding categories.

### 2.5. Study Selection Procedure

Study selection adhered to a three-stage PRISMA 2020–guided screening protocol to ensure transparency, replicability, and consistency in decision-making. The complete study flow, including identification, screening, eligibility assessment, and final inclusion, is presented in Figure 1, and the full PRISMA 2020 compliance checklist is provided in Supplementary File S1.

Stage 1: Title–Abstract Screening

All retrieved records were independently screened by two reviewers at the title–abstract level. To minimize false negatives and premature exclusion, studies presenting conceptual or methodological ambiguity were provisionally retained for full-text evaluation.

Stage 2: Full-Text Screening

Full manuscripts were evaluated against predefined eligibility criteria, with particular attention to:

(a) explicit AI-mediated feedback interaction,
(b) the presence of trace-based process indicators, and
(c) methodological adequacy for process extraction and analysis.

Exclusion decisions were documented systematically to ensure auditability and procedural transparency.

Stage 3: Reliability Assessment

Inter-rater agreement demonstrated strong consistency across screening stages (Title–Abstract: κ = 0.82; Full-Text: κ = 0.87), indicating substantial to near-perfect agreement and reinforcing the stability of inclusion decisions. Following duplicate removal and multi-stage screening, 33 studies met all eligibility criteria and were retained for final synthesis.

### 2.6. Corpus Overview: The 33 Included Studies

The final corpus comprised 33 empirically grounded investigations spanning diverse educational contexts, AI feedback architectures, and process-tracing methodologies. Collectively, these studies form a methodologically heterogeneous yet conceptually coherent evidence base, enabling comparative analysis of temporal, behavioral, and cognitive indicators within AI-mediated L2 writing environments. Table 3 summarizes the structural composition of the included corpus, including study design, feedback modality, trace methodology, and analytic focus.

This distribution illustrates both technological diversity and methodological heterogeneity across the dataset. To enhance corpus traceability, reproducibility, and alignment with the PRISMA 2020 reporting recommendations, a complete study characteristics matrix is provided in Appendix C, while the full extraction spreadsheet with all coded variables and study-level data is available in Supplementary File S3. The appendix reports authorship, participant characteristics, instructional context, AI feedback tool, feedback type, process-tracing methodology, and the principal temporal, cognitive, or behavioral indicators extracted for synthesis. An Evidence Mapping Matrix linking each included study to the three research questions and principal synthesis themes is provided in Appendix B.

#### 2.6.1. Coding of Process-Tracing Indicators

To systematize heterogeneous trace evidence, a hybrid deductive–inductive coding framework was developed as detailed in Table 4. Deductive categories were grounded in writing-process theory (Flower & Hayes, 1981), cognitive load theory, and feedback-uptake taxonomies, while inductive refinements emerged through iterative analysis of the included studies. Four domains structured the coding scheme. Temporal Dynamics (T-codes) captured pausing, burst writing, feedback latency, and revision duration. Cognitive–Metacognitive Processes (C-codes) included markers for indexed monitoring, evaluation of AI feedback, problem detection, and decision-making. Behavioral Feedback Engagement (B-codes) differentiated patterns of acceptance, modification, rejection, clarification queries, and recursive refinement. Methodological Rigor (M-codes) assessed logging precision, AI version transparency, triangulation, and analytic reproducibility. This architecture enabled the synthesis of diverse tracing methodologies within a theoretically aligned and analytically coherent framework.

To enhance coding transparency and illustrate how raw process-tracing observations were transformed into analytic categories, Table 5 presents representative examples of coding decisions from the included studies. The complete coding manual, operational definitions, and coding templates are provided in Supplementary File S4.

#### 2.6.2. Methodological Quality Coding

Because process-tracing studies differ widely in technical precision, an additional methodological coding layer was applied. This layer captures the structural integrity of trace data, something standard systematic reviews cannot evaluate. Table 6 summarizes the methodological quality indicators for process-tracing evidence for this study.

#### 2.6.3. Reliability of Extraction and Coding

To ensure coding stability and evidentiary consistency, 25% of the dataset was independently coded by two researchers across all analytic layers: categorical metadata, process-tracing indicators, behavioral uptake classifications, and methodological quality markers. Inter-coder agreement demonstrated strong to near-perfect reliability (Cohen’s κ = 0.91 for categorical data; κ = 0.88 for process indicators; κ = 0.86 for behavioral uptake codes; κ = 0.84 for methodological quality codes), supporting the robustness of the extraction framework. Discrepancies were resolved through structured discussion, and the coding manual was iteratively refined to enhance clarity, definitional precision, and replicability.

### 2.7. Risk of Bias and Methodological Quality Appraisal

**Figure 2:**
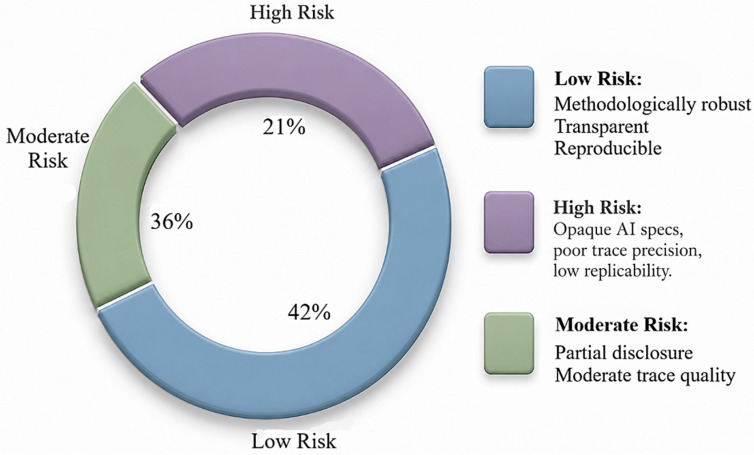
Risk-of-Bias Distribution Across Included Studies.

Methodological quality was assessed using the Mixed Methods Appraisal Tool MMAT to accommodate the quantitative, qualitative, and mixed-methods designs represented in the corpus. As measured in Figure 2, each study was evaluated for design appropriateness, sampling adequacy, data-collection integrity, analytic rigor, and coherence between the evidence and the interpretation. Given the technical demands of real-time process-tracing research, additional domain-specific criteria were applied to assess trace precision (e.g., timestamp granularity), instrumentation calibration, AI system transparency (model version and parameter disclosure), analytic reproducibility, and completeness of log data. Appraisals were conducted independently by two reviewers, with disagreements resolved through consensus.

Quality patterns were synthesized descriptively rather than reduced to aggregate scores, preserving methodological nuance. While several studies demonstrated high technical rigor and multimodal triangulation, substantial variability was observed in AI transparency, temporal resolution, and reproducibility reporting, factors that were considered when interpreting the strength of the cumulative evidence base.

Although methodological quality was appraised using MMAT and domain-specific trace-precision criteria, a full GRADE downgrading protocol was not implemented due to methodological heterogeneity; an adapted certainty-of-evidence framework was applied post hoc to evaluate cumulative confidence across research questions (see Section 3.7).

#### Study-Level Risk-of-Bias Summary

To enhance transparency and align with PRISMA 2020 recommendations for study-level appraisal reporting, Table 7 presents a structured summary of risk-of-bias judgments across the 33 included studies using MMAT criteria and domain-specific trace-quality indicators. Ratings were assigned independently by two reviewers and resolved by consensus.

Overall, 14 studies (42%) were classified as methodologically robust (low risk across ≥4 domains), 12 (36%) demonstrated moderate risk patterns, and 7 (21%) exhibited substantial limitations, primarily due to missing AI version disclosure and insufficient temporal precision. Because the corpus comprised heterogeneous methodologies and did not involve pooled intervention effect sizes, funnel plot or Egger regression analyses were not appropriate. To mitigate reporting bias, grey literature, dissertations, and conference proceedings were included. No evidence of systematic suppression of null findings was identified, although selective reporting of inferential statistics in primary studies cannot be ruled out.

## 3. Results

This section reports findings structured explicitly around the three research questions. Because the 33 included studies differed in design (quantitative = 15; qualitative = 8; mixed methods = 10), tracing modality (keystroke logging = 14; eye-tracking = 4; screen capture/logs = 9; multimodal = 6), and AI type (AWE = 18; LLM-based = 10; hybrid AI–human = 5), a configurative synthesis with descriptive quantification was conducted rather than a pooled meta-analysis. Across all analyses, frequencies are reported at the study level (k = number of studies; % = proportion of corpus).

### 3.1. RQ1: What Temporal Interaction Patterns Emerge When Learners Engage with AI-Assisted Feedback?

#### 3.1.1. Distribution of Temporal Evidence Across Studies

Across the 33 included studies, temporal evidence was unevenly distributed across tracing modalities. High-resolution keystroke logging was employed in 14 studies (42%), making it the dominant process-tracing method, while eye-tracking appeared in only 4 studies (12%). Nine investigations (27%) relied primarily on timestamped interaction or draft-history logs, and 6 (18%) implemented multimodal triangulation combining logs with complementary data sources. Notably, only 12 studies (36%) reported millisecond-level timestamp precision sufficient for fine-grained latency modeling, indicating that although temporal tracing is increasingly adopted, true high-resolution temporal analysis remains methodologically limited within the current evidence base.

#### 3.1.2. Immediate Uptake Loops in Micro-Level Editing

Across 24 of 33 studies (73%), micro-level feedback (grammar, spelling, lexical substitution) was associated with:

- Short latency windows (<5 s between feedback display and revision initiation)
- Clustered “burst” editing patterns
- High direct-acceptance rates (mean uptake range: 62–88% across AWE studies)
- Keystroke datasets showed:
- Mean pause duration before micro-revision: 1.8–3.2 s
- Burst lengths significantly longer immediately after AI feedback (Δ burst length = +23–41% relative to drafting baseline)

In 8 studies that reported inferential statistics, micro-level AI suggestions significantly increased revision frequency compared with baseline drafting (*p* < 0.05 across all reported models). The evidence suggests that AI feedback may function as a temporal accelerator for low-level edits, compressing revision windows and increasing revision density.

#### 3.1.3. Elongated Latency for Macro-Level Feedback

Macro-level feedback targeting coherence, argumentation, and structural organization exhibited substantially different temporal signatures from surface-level corrections. Across 19 studies (58%), the mean latency prior to macro-level revision ranged from 7 to 21 s, with recursive returns to the same text segment occurring in approximately 63% of macro-coded revisions. Eye-tracking evidence further indicated significantly longer fixation durations on global feedback (34–52% longer than micro-level suggestions), suggesting intensified evaluative processing. In four studies that reported inferential comparisons, decision cycles for macro-level feedback were significantly longer than those for surface corrections (*p* < 0.01). Across comparable datasets, macro-level latency was approximately 3.2–4.8 times longer than micro-level latency, reinforcing the interpretation that higher-order feedback imposes greater cognitive load and requires deeper interpretive engagement.

#### 3.1.4. Hybrid AI-Human Temporal Fragmentation

In the five hybrid AI–human studies (15% of the corpus), a consistent sequencing pattern emerged: AI-assisted surface revision preceded teacher-provided macro-level feedback in four cases, while three studies documented renewed AI polishing cycles following instructor input. Temporal modeling indicated an average of two to three oscillatory revision cycles per assignment, with re-entry latency decreasing across successive cycles, suggesting gradual trust calibration and procedural stabilization. Importantly, hybrid configurations did not shorten overall revision time; instead, they redistributed cognitive and temporal effort between human and AI agents, reflecting a reallocation of revision workload rather than a reduction in it.

#### 3.1.5. Minimal-Engagement Profiles

Six studies (18% of the corpus) identified shallow-engagement temporal signatures marked by near-zero latency acceptance (<1 s), reduced fixation durations, full-acceptance rates exceeding 90%, and minimal recursive checking of revised segments. In three quantitative investigations, these low-regulation profiles were significantly associated with lower language proficiency, weaker feedback literacy, and higher AI dependency (*p* < 0.05), indicating that rapid compliance with AI suggestions may reflect cognitive disengagement rather than efficient revision behavior.

#### 3.1.6. Synthesis for RQ1

Across the reviewed corpus (N = 33), a consistent temporal bifurcation pattern emerged in AI-mediated L2 revision. Process-tracing evidence suggests that micro-level revisions (e.g., lexical and surface-form edits) tend to cluster in rapid, burst-based sequences, whereas macro-level revisions (e.g., idea development, argument restructuring, discourse reorganization) are characterized by delayed, recursive engagement (Table 8). Rather than eliminating cognitive effort, AI appears to redistribute temporal attention across writing levels, shifting cognitive load toward higher-order evaluative processing.

AI reshapes revision not by eliminating effort but by redistributing temporal attention across levels of writing.

### 3.2. RQ2: What Cognitive, Metacognitive, and Behavioral Processes Are Inferable from Real-Time Traces?

#### 3.2.1. Cognitive Offloading

Across 17 studies (52% of the corpus), evidence pointed to cognitive offloading patterns in which learners accepted AI suggestions without systematic inspection, substituted AI-generated content for independent ideation during idea-generation tasks, and exhibited reduced monitoring behaviors, as inferred from limited gaze switching and minimal revision toggling. In five studies reporting correlational analyses, higher reliance on AI was negatively associated with independent revision effort (r = −0.32 to −0.51), and AI dependency significantly predicted reduced metacognitive commentary during stimulated recall protocols (*p* < 0.05), suggesting that increased automation may attenuate reflective oversight in the revision process.

#### 3.2.2. Deep Cognitive Engagement

In 12 studies (36%), typically involving advanced learners:

- Selective uptake was dominant (partial modification > direct acceptance);
- Fixation-switching between the AI suggestion and the original text indicated comparison behaviors;
- Rejection rates for macro suggestions ranged from 28–47%.

Three studies reported that proficiency significantly moderated uptake style (interaction effects *p* < 0.05).

#### 3.2.3. Behavioral Uptake Pathways

Table 9 shows behavioral uptake patterns, indicating structured variability in learners’ responses to AI-mediated feedback. Full acceptance was most frequent in grammar-focused AWE environments, whereas partial modification and selective rejection were more common in LLM-mediated or macro-level revision tasks that required interpretive judgment. Recursive feedback cycles emerged primarily in hybrid or generative AI contexts. Notably, macro-level feedback exhibited rejection rates two to three times higher than those of micro-level feedback, suggesting greater evaluative resistance when revisions implicate discourse structure or argumentation rather than surface accuracy.

Macro-feedback rejection rates were 2–3 times higher than micro-feedback rejection rates.

Table 10 synthesizes effect sizes reported across the 15 quantitative studies, providing a comparative overview of magnitude, directionality, and distribution of observed effects. Overall, the evidence indicates moderate-to-large positive impacts of AI on micro-revision acceleration and text quality, alongside moderate negative associations between AI reliance and independent metacognitive regulation, suggesting a performance–regulation trade-off within AI-mediated writing contexts.

Effect sizes indicate moderate-to-large impacts for micro-level acceleration and moderate negative associations between AI reliance and independent regulation.

#### 3.2.4. Cognitive Risk Patterns

Nine studies (27% of the corpus) explicitly identified cognitive risk patterns associated with AI-mediated revision, including voice homogenization, mechanical insertion of cohesive devices without rhetorical integration, and dependency on AI-generated ideas in place of independent ideation. In three quantitative investigations, heavy reliance on AI tools was significantly associated with lower originality or authorial distinctiveness scores (*p* < 0.05), suggesting that efficiency gains may, in some contexts, come at the expense of epistemic ownership and stylistic individuality.

**Figure 3:**
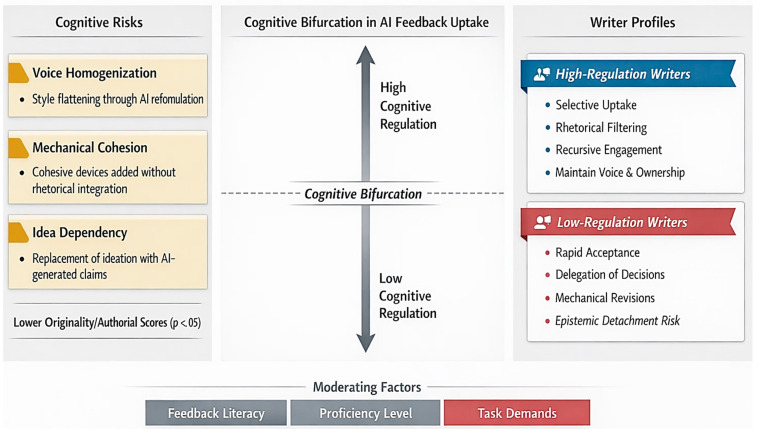
Cognitive Risk Patterns and Bifurcation Pathways in AI-Assisted Writing Revision.

Together, the evidence supports a cognitive bifurcation model of AI engagement. High-regulation writers indicate evaluative, selective, and recursive interaction with feedback, maintaining metacognitive oversight and rhetorical control. In contrast, low-regulation writers exhibit rapid acceptance patterns and cognitive delegation, often outsourcing both micro- and macro-level decisions to AI systems (Figure 3). Across studies, proficiency and feedback literacy consistently moderate the depth and quality of cognitive engagement.

#### 3.2.5. Synthesis for RQ2

Across the reviewed studies, learner engagement with AI-mediated feedback appeared to follow two broad regulatory pathways. Higher-regulation learners typically engaged in metacognitive filtering characterized by monitoring, evaluation, selective uptake, and iterative refinement of AI suggestions. In contrast, lower-regulation learners more frequently demonstrated cognitive offloading, reflected in rapid acceptance, limited verification, and reduced evaluative processing. These patterns varied according to learner proficiency, feedback literacy, task demands, and AI tool characteristics. Overall, the evidence suggests that the educational value of AI feedback depends not only on the quality of the feedback itself but also on learners’ capacity to critically evaluate, regulate, and strategically incorporate AI-generated suggestions during the writing process.

### 3.3. RQ3: How Methodologically Robust Is the Current Process-Tracing Literature?

#### 3.3.1. Trace Precision

Methodological precision varied substantially across the corpus. As revealed in Table 11, only 12 studies (36%) provided millisecond-level timestamps adequate for fine-grained latency modeling, while three of the four eye-tracking studies (75%) reported proper calibration standards. AI system transparency was limited: only 9 studies (27%) disclosed model versions or parameters, and multimodal triangulation was used in only 6 investigations (18%).

#### 3.3.2. Analytic Sophistication

Analytic sophistication across the corpus was uneven, with substantial variation in the depth and rigor of methods used to interpret process-tracing data. While multiple analytic approaches were employed, they were applied inconsistently and often failed to fully exploit temporal or sequential data structures. Table 12 summarizes the distribution of analytic methods and their principal limitations.

Only 5 studies (15%) reported reproducible analytic pipelines with scripts or transparent modeling steps.

#### 3.3.3. Structural Gaps

**Figure 4:**
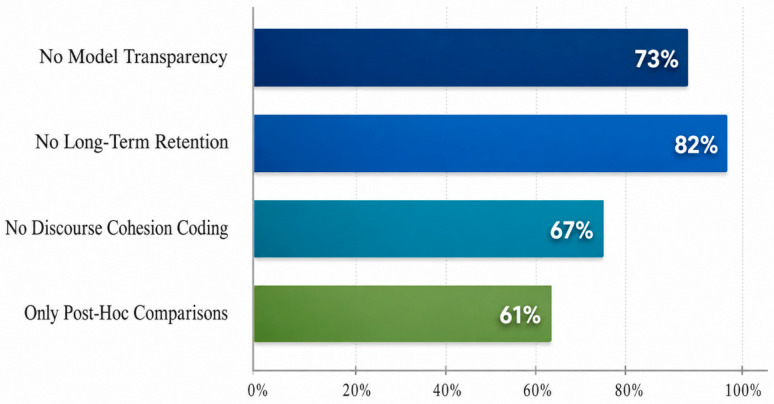
Structural Limitations Undermining Transparency and Methodological Rigor in AI-Mediated Writing Research.

Across the corpus, several structural limitations constrained cumulative interpretability and reproducibility. A substantial majority (73%) of studies did not disclose AI model versions or parameter settings, undermining transparency in rapidly evolving system environments, as shown in Figure 4. Longitudinal retention or transfer measures were absent in 82% of investigations, limiting insight into durable learning effects beyond immediate revision cycles. Additionally, 67% of studies did not operationalize cohesion at the discourse level through systematic coding of referential ties or rhetorical structure, and 61% relied primarily on post hoc draft comparisons rather than on genuine real-time process modeling. Collectively, these gaps highlight methodological fragmentation and the need for standardized reporting and tracing protocols.

### 3.4. Synthesis for RQ3

Overall, the field reflects notable conceptual innovation alongside pronounced technical fragmentation. High-quality investigations suggest the feasibility of multimodal tracing architectures, fine-grained latency modeling, and cognitive triangulation integrating behavioral, gaze-based, and self-report data. However, the absence of standardized temporal coding frameworks and consistent transparency in AI systems, including versioning and parameter disclosure, substantially constrains cross-study comparability, reproducibility, and cumulative theory-building in AI-assisted writing research.

### 3.5. Integrated Cross-RQ Statistical Overview

Across the three research questions, a coherent quantitative pattern emerges. A substantial majority of studies (73%) report accelerated micro-revision cycles, while 58% document delayed macro-level processing indicative of deeper interpretive engagement. Cognitive offloading patterns are identified in 52% of the corpus, whereas only 36% suggest sustained high-level evaluative filtering characteristic of metacognitively regulated engagement. Notably, just 15% of studies meet high reproducibility standards for trace precision, analytic transparency, and AI version reporting, underscoring a significant methodological gap despite strong empirical momentum.

### 3.6. Certainty of Evidence Assessment

Although the review employed a configurative synthesis approach, an adapted GRADE-style framework was applied post hoc to assess the overall confidence in the cumulative findings across the research questions. Evidence certainty was evaluated based on methodological consistency, trace precision, replication frequency, and risk-of-bias distribution as detailed in Table 13.

Evidence for temporal bifurcation patterns is supported by replication across 73% of studies but is limited by heterogeneity in trace precision. Cognitive offloading findings are consistent but partially dependent on correlational designs. Methodological fragmentation findings demonstrate high certainty due to the frequency of direct reporting and minimal interpretive inference.

### 3.7. Sensitivity Analysis

To assess the robustness of synthesis findings, analyses were re-conducted, excluding the seven studies classified as high risk of bias. The removal of these studies did not substantially alter the core patterns. Temporal bifurcation remained evident (micro-acceleration reported in 71% vs. original 73%), and cognitive offloading patterns remained identifiable (49% vs. original 52%). Effect directions and interpretive conclusions were therefore stable under quality-restricted conditions, suggesting that principal findings are not driven by lower-quality investigations.

## 4. Discussion

This review synthesizes process-tracing evidence to explain how AI reorganizes revision behavior, cognitive regulation, and construct representation during L2 writing, thereby shifting attention from product-based outcomes to the mechanisms through which learners engage with AI-mediated feedback. Across the corpus, three interconnected patterns emerge: temporal redistribution of revision effort, divergence between regulated filtering and delegative uptake, and substantial methodological fragmentation in tracing AI-mediated interaction. Collectively, the findings reposition AI-assisted writing as a distributed human–AI regulatory system rather than a purely outcome-oriented instructional intervention.

### 4.1. AI Reorganizes Rather than Reduces Revision Effort

A consistent pattern across 73% of studies indicates that AI accelerates micro-level revisions while elongating macro-level deliberation. Surface corrections (grammar, lexis, phrasing) are typically adopted within seconds, producing burst-like editing sequences. In contrast, discourse-level feedback addressing coherence, argumentation, and rhetorical structure triggers longer latency windows and recursive revisiting of text segments.

This bifurcation refines earlier findings that automated feedback increases revision frequency (Stevenson & Phakiti, 2019; Ranalli, 2018). Rather than uniformly reducing cognitive effort, AI appears to redistribute effort across levels of writing. Lower-level linguistic decisions become partially automated, whereas higher-order rhetorical decisions remain cognitively demanding. From a cognitive load perspective (Sweller et al., 2019), AI reduces extraneous load for surface-level monitoring but may simultaneously increase germane load when learners must evaluate global coherence or negotiate stylistic intent. Importantly, this pattern complicates simplistic claims that AI “makes writing easier.” Instead, writing becomes temporally asymmetric: compressed at the micro level and expanded at the macro level.

### 4.2. Divergent Cognitive Pathways: Filtering Versus Offloading

The most theoretically consequential finding concerns the divergence between metacognitive filtering and cognitive offloading. Approximately half of the reviewed corpus exhibited automation-driven acceptance behaviors, characterized by rapid uptake, limited gaze switching, and minimal revision toggling. In contrast, a smaller subset of studies documented selective modification, rejection, and recursive re-engagement behaviors indicative of active monitoring and evaluative reasoning. This divergence resonates with feedback literacy research (Carless & Boud, 2018), which emphasizes that feedback’s impact depends not on its availability but on learners’ interpretation and regulation. Collectively, these patterns support a bifurcation model of AI-mediated cognitive engagement in which learners diverge into distinct regulatory pathways during revision.

Process-tracing evidence supports a bifurcation pattern in AI-mediated revision. High-regulation writers engage in selective filtering, recursive evaluation, and rhetorical negotiation of AI suggestions, whereas low-regulation writers exhibit rapid uptake and delegative revision behavior. The negative association between AI reliance and metacognitive indicators (r = −0.32 to −0.51) suggests that performance gains may coexist with reduced independent monitoring. From a construct validity perspective, sustained AI mediation may shift the interpretation of assessments away from independent writing competence toward hybrid human–AI performance regulation.

#### 4.2.1. An Integrative Human–AI Revision Regulation Framework

**Figure 5:**
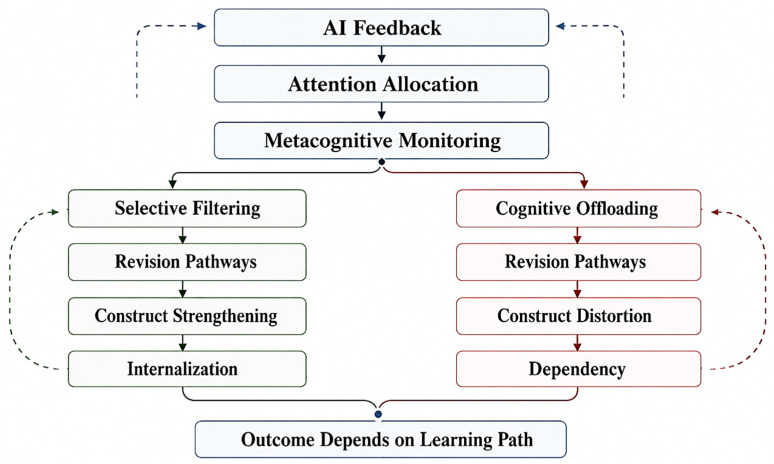
Integrative Human–AI Revision Regulation Framework for AI-Mediated L2 Writing.

To synthesize the cognitive and validity-related patterns identified across the reviewed studies, this review proposes an Integrative Human–AI Revision Regulation Framework (Figure 5). The framework conceptualizes AI-assisted revision as a conditional regulatory process in which learners evaluate, negotiate, or delegate writing decisions as they interact with AI-generated feedback.

The framework begins with AI feedback exposure, which directs learners’ attention to the linguistic, rhetorical, and organizational features of writing. Learners then engage in metacognitive monitoring by evaluating, verifying, and judging AI-generated suggestions. Revision behavior subsequently diverges into two pathways: selective filtering and cognitive offloading. Selective filtering involves critically evaluating and adapting AI suggestions while maintaining rhetorical control, whereas cognitive offloading involves rapid acceptance of AI recommendations with limited evaluative monitoring.

These pathways shape construct representation differently. Selective filtering is associated with deeper engagement and stronger alignment between writing performance and underlying competence, while cognitive offloading may distort construct representation when AI-generated content substitutes for independently regulated composing processes. Over time, selective filtering may support internalization of revision strategies and metacognitive regulation, whereas persistent cognitive offloading may reinforce AI dependency. Collectively, the framework shifts attention from whether AI improves writing to how AI reorganizes attention, regulation, revision behavior, and construct representation across different learner and task conditions.

#### 4.2.2. Boundary Conditions and Inferential Limits of the Framework

The proposed framework should be interpreted as a conditional, context-sensitive model rather than as a universally stable account of AI-mediated writing behavior. Several boundary conditions constrain its applicability and interpretive strength.

To start with, the framework is most strongly supported in formative, low- to moderate-stakes revision contexts. Its applicability to high-stakes assessment settings remains less certain because time pressure, accountability demands, and independent-authorship requirements may alter revision behavior. In addition, evidence varies across feedback types. Consistent uptake patterns are more evident in grammar-focused automated feedback, whereas discourse-level generation and generative co-authorship involve greater cognitive negotiation and variability. Findings from automated corrective feedback should therefore not be generalized directly to unrestricted collaboration with generative AI.

Furthermore, most studies examined immediate revision behavior rather than delayed transfer, retention, or independent post-AI performance. Consequently, claims regarding internalization remain theoretically plausible but empirically provisional. Thus, the framework is supported more strongly by process-sensitive indicators, such as uptake behavior and revision patterns, than by direct evidence of internal cognitive states, which are measured only inferentially. Finally, variability across AI systems, prompting conditions, and interface designs limits strong generalization. The framework should therefore be interpreted as a mechanism-oriented model grounded in current evidence rather than a technologically invariant account of human–AI writing interaction.

### 4.3. Uptake Patterns and Revision Ecologies

Uptake behavior varied systematically across revision levels. Full acceptance predominated in grammar-focused AWE contexts, whereas discourse-level suggestions elicited substantially higher rates of partial modification and selective rejection. Macro-level revision, therefore, appeared less automation-compatible and more dependent on interpretive negotiation aligned with rhetorical intent and task demands.

The emergence of recursive feedback cycles in hybrid AI–human contexts suggests the formation of a distributed revision ecology. In these environments, learners oscillate between AI-generated surface refinement and human-mediated rhetorical development. Such patterns support sociocognitive models of writing (Flower & Hayes, 1981; Révész et al., 2019), in which composing is distributed across cognitive agents and artifacts rather than confined to individual cognition.

### 4.4. Ethical and Construct Implications

Beyond behavioral dynamics, the review highlights ethical and epistemic risks. Several studies reported voice homogenization, stylistic flattening, and diminished authorial distinctiveness when AI-generated phrasing was adopted wholesale. These concerns echo broader critiques of generative AI in education (Kasneci et al., 2023; Godwin-Jones, 2024). Crucially, the risk appears conditional rather than universal. In contexts where learners lack AI literacy instruction, revision may shift from reflective problem-solving to optimization of machine-generated output. However, in environments that emphasize justification, attribution, and critical interrogation of AI feedback, such risks appear to be mitigated. Thus, AI’s pedagogical impact depends less on tool capability than on instructional framing.

More fundamentally, the evidence suggests that AI-assisted writing may increasingly instantiate a hybrid construct involving linguistic competence, evaluative regulation, feedback literacy, and human–AI orchestration ability.

#### Ethical and Data-Governance Implications

Process-tracing research generates highly granular behavioral data, including keystrokes, revision histories, interaction logs, screen recordings, and eye-tracking records. While these data provide valuable insights into learner cognition and feedback engagement, they also raise important concerns regarding privacy, informed consent, anonymization, storage security, and data sharing. Across the reviewed studies, ethical approval and participant consent were commonly reported, but detailed information on data retention policies, access controls, anonymization procedures, and long-term governance of trace data was often limited. These concerns are amplified in AI-mediated environments, where learner data may be processed through external platforms and cloud-based systems. Future research should adopt more transparent reporting of consent procedures, data-protection measures, storage practices, and sharing protocols to strengthen participant protection, reproducibility, and trust in AI-assisted writing research.

### 4.5. Methodological Fragmentation and the Limits of Current Evidence

Despite conceptual momentum, the review reveals substantial technical fragmentation. Only 36% of studies used millisecond-level timestamps, 27% disclosed AI model versions, and fewer than 20% implemented multimodal triangulation. These limitations constrain interpretive confidence, particularly regarding fine-grained latency modeling and causal inference. The absence of consistent AI parameter reporting (e.g., model versions, temperature settings) is particularly concerning. In rapidly evolving LLM environments, replicability depends on transparent specification. Without such documentation, cross-study comparisons risk conflating technological variance with learner behavior. Furthermore, most studies operationalize cohesion and coherence descriptively rather than through systematic discourse-analytic coding. Without standardized micro- and macro-level measures, claims regarding rhetorical development remain impressionistic.

### 4.6. Advancing a Process-Tracing Framework for AI-Assisted Feedback (PTF-AF)

In response to these gaps, the proposed Process-Tracing Framework for AI-Assisted Feedback (PTF-AF) emphasizes four principles:

1. Temporal granularity: Fine-grained logging of pauses, bursts, and latency to identify decision thresholds.
2. Cognitive triangulation: Integration of behavioral traces with stimulated recall and gaze-based attention indicators.
3. AI transparency: Systematic reporting of model versioning, parameterization, and update cycles.
4. Discourse anchoring: Standardized coding of referential cohesion and rhetorical structure to link process and product.

This framework shifts the field from outcome evaluation toward mechanism-sensitive inquiry. Rather than asking whether AI improves writing, future research should examine how AI reorganizes decision-making, agency, and the representation of constructs across developmental stages.

## 5. Conclusions

This PRISMA-guided systematic review synthesizes real-time process-tracing evidence to clarify how AI-mediated feedback reshapes L2 writing. Across the corpus, three stable patterns emerge. First, AI reorganizes the temporal architecture of revision: micro-level corrections are accelerated, whereas macro-level rhetorical decisions require sustained deliberation. Writing effort is redistributed rather than reduced. Second, learner engagement can be divided into two cognitive pathways: metacognitive filtering and cognitive offloading. While strategically regulated writers use AI dialogically, less regulated writers are more prone to automation-driven delegation, raising concerns about the representation of constructs. Third, the methodological infrastructure of the field remains fragmented, with limited standardization in trace precision, AI transparency, and discourse-level measurement.

Together, these findings reposition AI-assisted writing as a process-sensitive phenomenon embedded within evolving human-AI cognitive systems. The central question is no longer whether AI improves writing outcomes but how it reorganizes decision-making, agency, and the enactment of constructs. Future research should therefore investigate the developmental conditions under which learners move from cognitive offloading toward metacognitive filtering, particularly across differences in proficiency, feedback literacy, and AI familiarity. Accordingly, AI-assisted writing should be understood not simply as a technologically enhanced composition, but as a temporally evolving human–AI cognitive system in which authorship, regulation, and rhetorical control are continuously renegotiated during the act of writing itself. Advancing the field will require temporally granular designs, multimodal triangulation, systematic reporting of AI configurations, and theoretically anchored models of engagement. Only through such methodological consolidation can research move beyond outcome comparisons toward a mechanism-level understanding of AI-mediated writing development.

### 5.1. Limitations of the Present Review

The present synthesis is constrained by substantial methodological heterogeneity across primary studies. Variation in trace granularity, AI system architecture (rule-based AWE vs. LLMs), discourse operationalization, and analytic pipelines limited the feasibility of pooled meta-analysis and necessitated a configurative approach. As systematic review methodology cautions, high structural heterogeneity reduces statistical comparability and shifts interpretation toward pattern convergence rather than effect precision (Petticrew & Roberts, 2006; Higgins et al., 2024).

Although an adapted certainty-of-evidence framework was applied, a full GRADE downgrading protocol was not implemented due to the corpus’s mixed-methods and mechanism-oriented design. Many cognitive offloading findings derive from correlational analyses, which, while consistent, do not establish directional causality (Shadish et al., 2002). Thus, interpretations of delegation and monitoring attenuation remain theoretically grounded but inferential. Technological volatility further constrains generalization. AI systems evolve rapidly, and only a minority of included studies reported model versions or parameter configurations. Given concerns regarding model drift and replicability in educational AI research (Kasneci et al., 2023; Godwin-Jones, 2024), observed behavioral patterns should be interpreted as temporally situated rather than technologically invariant.

Process-tracing methods provide powerful behavioral indicators but remain inferential proxies for cognition. Pauses, fixations, and revision bursts approximate planning and monitoring processes but do not directly measure metacognitive states (Leijten & Van Waes, 2013; Révész et al., 2019). While grey literature was incorporated to mitigate reporting bias, selective publication effects cannot be entirely ruled out (Higgins et al., 2024). Accordingly, findings should be interpreted as high-confidence syntheses within documented methodological and technological boundaries rather than definitive causal models.

### 5.2. Theoretical Implications

The findings advance L2 writing theory in three respects. First, they conceptualize AI-mediated revision as temporally bifurcated rather than uniformly accelerated. Second, they formalize a bifurcation model of cognitive engagement, distinguishing filtering from delegation. Importantly, this bifurcation model provides a theoretically expandable architecture for future research by generating testable pathways linking AI reliance, metacognitive regulation, feedback literacy, proficiency, and construct representation. In this sense, the framework functions not merely as a descriptive typology, but as a mechanism-oriented model of human–AI cognitive interaction during writing. Third, they foreground concerns about the representation of constructs in AI-assisted assessment contexts. Together, these contributions reposition AI not as an external enhancement tool but as an embedded cognitive agent that reshapes how writing ability is enacted, distributed, and interpreted.

## Figures and Tables

**Table 1:** Structured Keyword Clusters and Representative Search Terms for Database Retrieval.

Cluster	Examples
AI Feedback	“Automated writing evaluation”, AWE, AWCF, “AI feedback”, GPT, “large language model”, “neural feedback”
Process Tracing	“Keystroke logging”, “eye-tracking”, “screen capture”, “process mining”, “revision logs”, “timestamped analytics”
Writing Context	“L2 writing”, ESL, EFL, “revision behaviors”, “academic writing”

**Note:** AI = Artificial Intelligence; AWE = Automated Writing Evaluation; AWCF = Automated Writing Corrective Feedback; GPT = Generative Pre-trained Transformer; ESL = English as a Second Language; EFL = English as a Foreign Language.

**Table 2:** Comprehensive Data Extraction Matrix for Process-Tracing Studies on AI-Assisted Feedback.

Extraction Category	Operational Definition	Examples/What Was Extracted
Study Identification	Bibliographic metadata	Authors, year, title, country, journal/conference
Participant Characteristics	Demographics and proficiency	Sample size, L1, L2 level (CEFR, TOEFL), education level
Writing Tasks/Genres	Nature of writing produced	Argumentative essays, summaries, email writing, and timed tasks
AI Tool Description	Type and features of AI feedback used	GPT-based tools, AWE systems, grammar checkers, and feedback categories (grammar, style, cohesion)
Human Feedback Description	If applicable, teacher/peer involvement	Peer comments, teacher annotations, hybrid cycles
Process-Tracing Method	The tracing technology used	Keystroke logging, eye-tracking, screen capture, and event logs
Temporal Indicators	Micro-temporal behavioral signals	Pauses (>2 s), bursts, revision latency, first-pass reading time
Cognitive/Metacognitive Indicators	Behaviors reflecting reasoning or monitoring	Fixation durations, regressions, toggling behaviors, and self-repair patterns
Revision Behaviors	Nature and depth of revisions	Micro vs. macro revisions, feedback uptake types, restructuring
AI Feedback Interactions	How learners engaged with AI suggestions	Acceptance, rejection, selective modification, querying
Analytical Techniques	Process-level analytic pipelines	Process mining, sequence analysis, heatmaps, transition matrices
Validity/Reliability Indicators	Technical and methodological rigor	Timestamp alignment, tool calibration, inter-rater checks
Ethical Considerations	Handling of sensitive log data	Consent, storage protocols, anonymization
Quantitative Outcomes	Numeric/traced results	Pause distributions, fixation counts, revision frequencies
Qualitative Outcomes	Interpretive insights	Learner explanations, perceived usefulness/overload
Limitations Identified	Author-reported shortcomings	Missing logs, incomplete traces, and limited generalizability

**Table 3:** Overview of Included Studies (N = 33).

Dimension	Distribution
Total Studies	33
Quantitative	15
Qualitative	8
Mixed-Methods	10
Keystroke Logging	14
Eye-Tracking	4
Screen Capture/Interaction Logs	9
Hybrid/Multi-modal Tracing	6
AWE Systems	18
LLM-Based Feedback	10
Hybrid AI–Human Feedback	5

**Table 4:** Coding Framework for Temporal, Cognitive, and Behavioral Indicators.

Domain	Code	Definition	Examples in Trace Data
**Temporal**	T1	Pauses	Pauses > 2000 ms before interacting with AI feedback; hesitation before revising
	T2	Bursts	Rapid sequences of typing following AI suggestion acceptance
	T3	Latency	Time between AI feedback appearance and learner action
	T4	Revision Duration	Length of time spent revising each unit of text
**Cognitive/Metacognitive**	C1	Monitoring	Fixations on AI feedback, repeated viewing of the suggestion
	C2	Evaluation	Comparison behaviors (fixation shifts between AI and learner text)
	C3	Problem Detection	Backspacing clusters, revision initiation markers
	C4	Decision-Making	Accept/reject patterns, toggling between versions
**Behavioral**	B1	Full Uptake	Direct acceptance via one-click adoption
	B2	Partial Uptake	Learner edits the AI suggestion before integrating
	B3	Rejection	No use of AI suggestion; retyping original content
	B4	Clarification Queries	Asking AI for alternative versions or explanations
	B5	Recursive Cycles	AI → revision → AI feedback → revision patterns

**Table 5:** Illustrative Examples of Coding Decisions from Included Studies.

Study	Extracted Trace Observation	Interpretation	Assigned Code
Ranalli (2018)	AI suggestion accepted without modification	Direct adoption of AI feedback	B1 (Full Uptake)
Koltovskaia (2020)	Repeated viewing of the AI suggestion before revision	Monitoring and evaluation behavior	C1 (Monitoring)
Tian et al. (2023)	8-s delay before initiating revision	Extended response latency	T3 (Latency)

**Table 6:** Methodological Quality Indicators for Process-Tracing Evidence.

Quality Dimension	Indicator	Criteria for High Quality
Trace Precision	Timestamp Integrity	Logs synchronized to milliseconds; no missing sequences
Tool Calibration	Sensor Accuracy	Eye-tracker calibrated to <0.5° visual angle; keystroke logger tested
AI Transparency	Versioning/Model Disclosure	AI tool version reported, update cycles documented
Triangulation	Multi-Modal Evidence	Combination of logs + eye-tracking + interviews
Analytic Reproducibility	Pipeline Documentation	Scripts, algorithms, or analysis steps provided
Data Completeness	Missing Data Handling	Missing fixations/log events reported and accounted for

**Table 7:** Study-Level Risk-of-Bias Assessment Across Included Studies (N = 33).

Quality Domain	Low Risk (n, %)	Moderate Risk (n, %)	High Risk (n, %)
Sampling Adequacy	21 (64%)	9 (27%)	3 (9%)
Design Appropriateness	25 (76%)	6 (18%)	2 (6%)
Trace Precision (timestamp integrity)	12 (36%)	15 (45%)	6 (18%)
AI Transparency (version disclosure)	9 (27%)	8 (24%)	16 (49%)
Analytic Rigor	17 (52%)	10 (30%)	6 (18%)
Data Completeness	19 (58%)	9 (27%)	5 (15%)

**Table 8:** Temporal Bifurcation in AI-Mediated Revision: Latency Patterns and Cognitive Signatures Across Writing Levels.

Revision Type	Latency Pattern	Cognitive Signature
Micro-level	Rapid, burst-based	Low cognitive friction
Macro-level	Delayed, recursive	Deep evaluative processing

**Table 9:** Behavioral Uptake Pathways in AI-Mediated L2 Writing: Reporting Frequency and Contextual Distribution Across Studies.

Uptake Type	Studies Reporting	Typical Context
Full Acceptance	26 (79%)	Grammar-focused AWE
Partial Modification	18 (55%)	LLM or macro tasks
Selective Rejection	14 (42%)	Coherence/argument feedback
Recursive Cycles	11 (33%)	Hybrid or LLM contexts

**Table 10:** Summary of Reported Effect Sizes in Quantitative Studies (k = 15).

Outcome Type	Effect Size Range	Direction	Studies Reporting
Micro-revision frequency	d = 0.42–0.81	Positive	6
Latency difference (macro vs. micro)	d = 0.55–1.02	Longer macro latency	4
AI vs. metacognitive monitoring	r = −0.32 to −0.51	Negative association	5
Uptake moderation by proficiency	η^2^ = 0.08–0.19	Moderate interaction	3
AI-assisted quality improvement	d = 0.38–0.74	Positive	7

**Table 11:** Proportion of Included Studies Meeting High Technical Standards.

Indicator	Studies Meeting High Standard
Millisecond timestamps	12 (36%)
Eye-tracker calibration reported	3 of 4 (75%)
AI version transparency	9 (27%)
Multimodal triangulation	6 (18%)

**Table 12:** Analytic Approaches Employed Across Included Studies and Associated Methodological Limitations.

Analytic Method	Studies Using	Limitations
Keystroke burst analysis	14	Often descriptive only
Process mining	2	No event-sequence graphs
Revision taxonomy coding	8	No cross-study standard
Psychometric modeling	10	Outcome-focused
Longitudinal follow-up	3	Limited transfer analysis

**Table 13:** Certainty-of-Evidence Matrix Across Research Questions.

Research Question	Consistency	Precision	Risk of Bias	Overall Certainty
RQ1: Temporal Bifurcation	High	Moderate	Moderate	Moderate–High
RQ2: Cognitive Offloading Patterns	Moderate	Moderate	Moderate	Moderate
RQ3: Methodological Fragmentation	High	High	Low Concern	High

## Data Availability

All data extracted and analyzed during this systematic review are derived from publicly available published studies cited in the reference list. The structured extraction matrix and coding framework were developed for this review and are available from the corresponding author upon reasonable request. No new primary datasets were generated.

## Supplementary Materials

The following supporting information can be downloaded at: https://www.mdpi.com/article/10.3390/bs16071229/s1, Supplementary File S1. Completed PRISMA 2020 Checklist; Supplementary File S2. Full Database Search Strategies; Supplementary File S3. Data Extraction and Coding Framework; Supplementary File S4. Risk-of-Bias and Methodological Quality Appraisal Framework.

## Appendix A

Database-Specific Boolean Search Strings for Retrieval of AI-Assisted Process-Tracing Studies in L2 Writing (2010–2025).

**Database**	**Search Field**	**Boolean Search String**	**Filters Applied**
**Scopus**	TITLE-ABS-KEY	TITLE-ABS-KE (“automated writing evaluation” OR AWE OR AWCF OR “automated written corrective feedback” OR “AI feedback” OR “generative AI” OR “large language model*” OR GPT OR “ChatGPT”) AND (“keystroke logging” OR “eye tracking” OR “process tracing” OR “process mining” OR “revision log*” OR “interaction log*” OR “draft history”) AND (“second language writing” OR “L2 writing” OR ESL OR EFL OR “academic writing”) AND PUBYEAR > 2009 AND PUBYEAR < 2026	2010–2025; English; Articles, Reviews, Conference Papers
**Web of Science Core Collection**	TS (Topic Search)	TS= (“automated writing evaluation” OR AWE OR AWCF OR “automated written corrective feedback” OR “AI feedback” OR “generative AI” OR “large language model*” OR GPT OR “ChatGPT”) AND (“keystroke logging” OR “eye tracking” OR “process tracing” OR “process mining” OR “revision log*” OR “interaction log*” OR “screen recording”) AND (“second language writing” OR “L2 writing” OR ESL OR EFL OR “academic writing”)	Timespan: 2010–2025; Indexes: SSCI, ESCI; English
**ERIC**	All Fields	(“automated writing evaluation” OR AWE OR AWCF OR “AI feedback” OR “generative AI” OR GPT) AND (“keystroke logging” OR “eye tracking” OR “writing process” OR “revision process” OR “interaction log*”) AND (“second language writing” OR ESL OR EFL)	Peer-reviewed; English; 2010–2025
**ProQuest**	NOFT (Anywhere Except Full Text)	NOFT (“automated writing evaluation” OR AWE OR AWCF OR “AI feedback” OR “large language model*” OR GPT OR “ChatGPT”) AND (“keystroke logging” OR “eye tracking” OR “process tracing” OR “process mining” OR “revision log*” OR “draft history”) AND (“second language writing” OR “L2 writing” OR ESL OR EFL))	2010–2025; English; Scholarly journals; Dissertations included

## Appendix B. Evidence Mapping Matrix

**Table A1:** Evidence Mapping Matrix Linking Included Studies to Research Questions.

Study	Primary Evidence Type	Evidence Mapping	RQ1	RQ2	RQ3
Barkaoui (2014)	Keystroke timing and writing-process measures	*	✓	**-**	✓
Li et al. (2015)	AWE uptake and revision behavior	*	✓	✓	✓
Liu and Kunnan (2015)	AWE revision patterns	*	✓	✓	**-**
Liao (2016)	Grammar correction and revision behavior	*	✓	✓	**-**
Chan (2017)	Keystroke-based process measures	*	✓	**-**	✓
Ranalli et al. (2017)	Feedback usefulness and uptake	*	✓	✓	✓
Ranalli (2018)	Acceptance, modification, and rejection of AWCF	*	✓	✓	✓
Smet et al. (2018)	Eye-tracking and keystroke process data	^	✓	✓	✓
Révész et al. (2019)	Pausing and revision behavior	^	✓	✓	✓
Koltovskaia (2020)	Engagement with Grammarly feedback	*	✓	✓	✓
Choi and Deane (2021)	Temporal writing-process indicators	*	✓	**-**	✓
Mohsen (2021)	Fluency and revision processes	*	✓	✓	✓
Ranalli (2021)	Trust and engagement with automated feedback	*	✓	✓	✓
Saricaoglu and Bilki (2021)	Voluntary AWE use patterns	*	✓	✓	**-**
Link et al. (2022)	Teacher–AI feedback interaction	*	✓	✓	✓
Ranalli and Yamashita (2022)	Feedback timing and correction performance	*	✓	✓	✓
Barrot (2023)	Accuracy gains and feedback uptake	*	✓	✓	
Guo et al. (2023)	Research-paper revision with AI feedback	*	✓	✓	✓
Lim et al. (2023)	Neural AWE feedback utilization	*	✓	✓	✓
Tian et al. (2023)	Argumentation and revision sequences	*	✓	✓	✓
Vandermeulen et al. (2023)	Process-feedback and synthesis writing	*	✓	✓	✓
T. Zhang and Mao (2023)	Feedback literacy development	^	**-**	✓	**-**
Zhu et al. (2023)	Editing and revision behavior	*	✓	✓	✓
Lin and Crosthwaite (2024)	Teacher vs. GPT feedback uptake	*	✓	✓	✓
Strobl et al. (2024)	ChatGPT-supported revision cycles	^	✓	✓	✓
Hwang et al. (2025)	Generative AI uses duration and patterns	*	✓	✓	✓
Mekheimer (2025)	Revision frequency and writing quality	*	✓	✓	**-**
Zhai (2025)	Self-regulation and motivational dynamics	^	✓	✓	**-**
Chan and Lam (2025)	Meaning-related revision processes	^	✓	✓	✓
Tian et al. (2025)	Keystroke-logging methodology and temporal indicators	*	✓	**-**	✓
Leijten and Van Waes (2013)	Keystroke-logging & writing-process visualization	^	✓	**-**	✓
Conklin and Pellicer-Sánchez (2016)	Eye-tracking for language-processing analysis	^	✓	**-**	✓
Révész et al. (2016)	Cognitive task-demand and process-tracing triangulation	^	✓	✓	✓

Note. * Indicates studies contributing to the synthesis reported in Section 3.1.2, in which 24 of 33 studies (72.7%, rounded to 73%) documented immediate uptake loops in micro-level editing. These studies reported evidence of local feedback or revision involving grammar, spelling, lexical substitution, corrective feedback, editing behavior, uptake, revision frequency, pause/burst patterns, system logs, usage logs, interaction logs, or draft-history records. Studies marked “^” were retained in the corpus but did not directly contribute to this specific 73% figure.

## Appendix C

**Table A2:** Characteristics of Included Studies.

Study	Sample	Context	AI Tool	Feedback Type	Tracing Method	Indicators
Barkaoui (2014)	TOEFL iBT test takers	Assessment writing	Writing platform	Writing-process analysis	Keystroke logging	Writing behaviors, fluency
Li et al. (2015)	University ESL learners	Academic writing	Criterion AWE	Automated feedback	Revision logs	Uptake, revision frequency
Liu and Kunnan (2015)	Chinese EFL majors	University writing	WriteToLearn	AWE feedback	Usage logs	Revision patterns
Liao (2016)	EFL undergraduates	Writing course	AWE platform	Grammar feedback	Draft-history analysis	Error correction, revision behavior
Chan (2017)	L2 test takers	Reading-to-write assessment	Writing platform	Writing-process analysis	Keystroke logging	Pauses, revisions
Ranalli et al. (2017)	L2 university writers	Academic writing	Criterion	Formative feedback	Interaction logs	Uptake, usefulness
Ranalli (2018)	L2 learners	Writing instruction	AWE	AWCF	Revision logs	Acceptance, modification, rejection
Smet et al. (2018)	Student writers	Writing research	Process-tracing environment	Process observation	Eye-tracking + keystroke logging	Fixations, pauses
Révész et al. (2019)	L2 writers	Writing tasks	Writing environment	Process baseline	Keystroke logging	Pausing behavior, revisions
Koltovskaia (2020)	EFL students	University writing	Grammarly	AWCF	Screen capture	Engagement, trust, uptake
Choi and Deane (2021)	Adult EFL writers	Assessment context	Writing evaluation system	Automated feedback	Keystroke logging	Pauses, bursts, fluency
Mohsen (2021)	L1/L2 writers	Writing comparison	Writing software	Process analysis	Keystroke logging	Fluency, revision behavior
Ranalli (2021)	L2 university students	Academic writing	AWE	Corrective feedback	Interaction logs	Trust, engagement
Saricaoglu and Bilki (2021)	Content-course students	Higher education	AWE	Voluntary feedback	Usage logs	Feedback utilization
Link et al. (2022)	ESL writers	Academic writing	AWE	Hybrid feedback	Revision records	Teacher–AI uptake
Ranalli and Yamashita (2022)	L2 writers	Writing instruction	AWCF system	Automated feedback	System logs	Timing, correction performance
Barrot (2023)	EFL learners	Writing classroom	AWCF system	Corrective feedback	Revision analysis	Accuracy, uptake
Guo et al. (2023)	Graduate writers	Research writing	AI feedback system	Revision feedback	Draft-history analysis	Feedback acceptance
Lim et al. (2023)	Korean L2 writers	Academic writing	Neural AWE	Automated feedback	System logs	Feedback use
Tian et al. (2023)	L2 writers	Argumentative writing	Writing analytics	Process feedback	Keystroke logging	Pauses, revision sequences
Vandermeulen et al. (2023)	Student writers	Synthesis writing	Process-feedback system	Keystroke-based feedback	Keystroke logging	Writing process
T. Zhang and Mao (2023)	L2 students	Writing classroom	Feedback tools	Feedback literacy	Classroom/process records	Evaluation, regulation
Zhu et al. (2023)	L2 learners	Academic writing	Process analytics	Revision support	Keystroke logging	Editing behavior
Lin and Crosthwaite (2024)	University L2 writers	Academic writing	ChatGPT	GPT-assisted feedback	Revision records	Uptake comparison
Strobl et al. (2024)	Advanced L2 writers	Writing class	ChatGPT	Generative feedback	Interaction logs	Prompting, revision cycles
Hwang et al. (2025)	L2 university writers	Academic writing	Generative AI	AI writing support	Interaction logs	Duration, revision behavior
Mekheimer (2025)	EFL students	Writing instruction	Generative AI	AI feedback	Revision records	Revision frequency, writing quality
Zhai (2025)	EFL learners	Academic writing	AWCF	Automated feedback	Process records	Self-regulation, motivation
Chan and Lam (2025)	L2 writers	Academic writing	Process analytics	Meaning-related revision	Keystroke logging	Meaning-related revisions
Tian et al. (2025)	L2 writers	Knowledge writing	KLIWC corpus	Process analysis	Keystroke logging	Bursts, pauses
Leijten and Van Waes (2013)	Student writers	Writing-process research	Inputlog	Process-tracing methodology	Keystroke logging	Writing-process metrics
Conklin and Pellicer-Sánchez (2016)	L2 learners	Applied linguistics	Eye-tracking environment	Process analysis	Eye tracking	Attention, reading behavior
Révész et al. (2016)	L2 learners	Task-based writing	Experimental tasks	Cognitive-demand analysis	Dual-task and self-report measures	Cognitive load, task demands
