# Supplementary material for: Metacognitive Filtering and Cognitive Offloading in AI-Assisted L2 Writing: A PRISMA Guided Process-Tracing Synthesis
Source: Behav Sci (Basel). 2026 Jul 20;16(7):1229. doi: 10.3390/bs16071229 (PMC13403878; doi:10.3390/bs16071229)
Supplement: Supplementary file 1 [file behavsci-16-01229-s001.zip › behavsci-4352844-supplementary.pdf]

Metacognitive Filtering and Cognitive Offloading in AI-Assisted L2 Writing: A PRISMA Guided Process-Tracing Synthesis

Supplementary File S1. PRISMA 2020 Checklist

PRISMA 2020 Checklist

Section and Topic	Item #	Checklist item (Official PRISMA 2020 wording)	Location where the item is reported
TITLE			
Title	1	Identify the report as a systematic review.	Title page; manuscript title
ABSTRACT			
Abstract	2	See the PRISMA 2020 for Abstracts checklist.	Abstract
INTRODUCTION			
Rationale	3	Describe the rationale for the review in the context of existing knowledge.	Sections 1.1–1.2 (Context and Significance; Problem Statement and Conceptual Gap)
Objectives	4	Provide an explicit statement of the objective(s) or question(s) the review addresses.	Section 1.3 Purpose and Contribution of the Study; Research Questions (RQ1–RQ3)
METHODS			
Eligibility criteria	5	Specify the inclusion and exclusion criteria for the review and how studies were grouped for the syntheses.	Section 2.4 Eligibility Criteria
Information sources	6	Specify all databases, registers, websites, organizations, reference lists, and other sources searched or consulted to identify studies. Specify the date when each source was last searched or consulted.	Section 2.3 Information Sources and Search Strategy
Search strategy	7	Present the full search strategies for all databases, registers, and websites, including any filters and limits used.	Section 2.3 Information Sources and Search Strategy; Table 1; Supplementary File S2
Selection process	8	Specify the methods used to decide whether a study met the inclusion criteria, including how many reviewers screened each record and report, whether they worked independently, and, if applicable, details of automation tools used in the process.	Section 2.5 Study Selection Procedure; Figure 1
Data collection process	9	Specify the methods used to collect data from reports, including how many reviewers collected data from each report, whether they worked independently, any processes for obtaining or confirming data from study investigators, and, if applicable, details of automation tools used in the process.	Section 2.4 Data Extraction Framework; Section 2.6.3 Reliability of Extraction and Coding
Data items	10a	List and define all outcomes for which data were sought. Specify whether all compatible results were sought and, if not, the methods used to decide which results to collect.	Section 2.4 Data Extraction Framework; Tables 2, 4, and 5
	10b	List and define all other variables for which data were sought (e.g., participant and intervention characteristics, funding sources). Describe any assumptions made about missing or unclear information.	Section 2.4 Data Extraction Framework; Tables 2 and 3
Study risk of bias assessment	11	Specify the methods used to assess the risk of bias in the included studies, including details of the tool(s) used, how many reviewers assessed each study, whether they worked independently, and, if applicable, details of the automation tools used in the process.	Section 2.7 Risk of Bias and Methodological Quality Appraisal; Figure 2; Table 7
Effect measures	12	Specify for each outcome the effect measure(s) (e.g., risk ratio, mean difference) used in the synthesis or presentation of results.	Sections 3.1–3.7
Synthesis	13a	Describe the processes used to decide which studies were eligible for each synthesis.	Sections 2.4–2.6

Section and Topic	Item #	Checklist item (Official PRISMA 2020 wording)	Location where the item is reported
methods	13b	Describe any methods required to prepare the data for presentation or synthesis, such as handling missing summary statistics or data conversions.	Sections 2.4 and 2.6
	13c	Describe any methods used to tabulate or visually display the results of individual studies and syntheses.	Tables 1–12; Figures 1–5
	13d	Describe any methods used to synthesize results and provide a rationale for the choice(s). If a meta-analysis was performed, describe the model(s), methods for identifying heterogeneity, and the software used.	Sections 2.1, 2.2, and Section 3
	13e	Describe any methods used to explore possible causes of heterogeneity among study results (e.g., subgroup analysis, meta-regression).	Sections 3.1–3.3
	13f	Describe any sensitivity analyses conducted to assess the robustness of the synthesized results.	Section 3.8 Sensitivity Analysis
Reporting bias assessment	14	Describe any methods used to assess the risk of bias due to missing results in a synthesis (arising from reporting biases).	Section 2.7 Risk of Bias and Methodological Quality Appraisal
Certainty assessment	15	Describe any methods used to assess certainty (or confidence) in the body of evidence for an outcome.	Section 2.7 Risk of Bias and Methodological Quality Appraisal; Section 3.7 Certainty of Evidence Assessment
Study selection			16a
Study characteristics	16b	Cite studies that appear to meet the inclusion criteria but were excluded, and explain why.	Figure 1: Section 2.5 Study Selection Procedure
	17	Cite each included study and present its characteristics.	Section 2.6 Corpus Overview; Table 3; Appendix C; Supplementary File S3
Risk of bias in studies	18	Present the risk-of-bias assessments for each included study.	Section 2.7 Risk of Bias and Methodological Quality Appraisal; Figure 2; Table 7
Results of individual studies	19	For all outcomes, present, for each study: (a) summary statistics for each group (where appropriate) and (b) an effect estimate and its precision, ideally using structured tables or plots.	Sections 3.1–3.7; Tables 8–12; Appendix C
Results of syntheses	20a	For each synthesis, briefly summarise the characteristics and the risk of bias of the contributing studies.	Sections 3.1–3.7
Reporting biases	20b	Present the results of all statistical syntheses conducted. If a meta-analysis was done, present the summary estimate, precision, and heterogeneity. If comparing groups, describe the direction of the effect.	Sections 3.1–3.7
	20c	Present the results of all investigations of possible causes of heterogeneity among study results.	Sections 3.3–3.5
	20d	Present the results of all sensitivity analyses conducted to assess the robustness of the synthesized results.	Section 3.8 Sensitivity Analysis.
	21	Present risk-of-bias assessments for missing results (arising from reporting bias) for each synthesis assessed.	Section 2.7 Risk of Bias and Methodological Quality Appraisal
Certainty of evidence	22	Present assessments of certainty (or confidence) in the body of evidence for each outcome assessed.	Section 3.7 Certainty of Evidence Assessment (supported by methods described in Section 2.7)

Section and Topic	Item #	Checklist item (Official PRISMA 2020 wording)	Location where the item is reported
Study selection	16a	Describe the results of the search and selection process, from the number of records identified to the number of studies included, ideally using a flow diagram.	Figure 1; Section 2.5 Study Selection Procedure
Discussion			23a
Registration and protocol	23b	Discuss any limitations of the evidence included in the review.	Section 4.2 Limitations
	23c	Discuss any limitations of the review processes used.	Section 4.2 Limitations
	23d	Discuss implications of the results for practice, policy, and future research.	Sections 4.3–4.7
	24a	Provide registration information for the review, including register name and registration number, or state that the review was not registered.	Section 2.2 Protocol and Reporting Standards
Other Information			24b
Support Competing interests	24c	Describe and explain any amendments to information provided at registration or in the protocol.	Section 2.2 Protocol and Reporting Standards
	25	Describe the sources of financial or non-financial support for the review and the role of the funders or sponsors.	Funding Statement
	26	Declare any competing interests of review authors.	Conflicts of Interest Statement
Availability of data, code and other materials	27	Report which of the following are publicly available and where they can be found: template data collection forms; data extracted from included studies; data used for all analyses; analytic code; any other materials used in the review.	Data Availability Statement; Supplementary Materials Statement; Supplementary Files S1–S4
Discussion	23a	Provide a general interpretation of the results in the context of other evidence.	Section 4 Discussion
	23b	Discuss any limitations of the evidence included in the review.	Section 4.2 Limitations

From: Page MJ, McKenzie JE, Bossuyt PM, Boutron I, Hoffmann TC, Mulrow CD, et al. The PRISMA 2020 statement: an updated guideline for reporting systematic reviews. BMJ 2021;372:n71. doi: 10.1136/bmj.n71.

This work is licensed under CC BY 4.0. To view a copy of this license, visit https://creativecommons.org/licenses/by/4.0/

Supplementary File S2. Full Database Search Strategies

S2.1 Search Design and Retrieval Framework

Search procedures were designed to identify empirical studies examining real-time process tracing in AI-assisted L2 writing environments. Search construction followed a concept-cluster approach integrating:

1. AI-mediated feedback systems,
2. process-tracing methodologies,
3. and L2 writing/revision contexts.

S2.2 Information Sources

The following databases and sources were systematically searched:

Source	Purpose
**Web of Science Core Collection**	Interdisciplinary indexed literature
**Scopus**	Broad multidisciplinary retrieval
**ERIC**	Education and applied linguistics research
**ProQuest Dissertations & Theses**	Grey literature retrieval
**Conference proceedings**	Emerging process-tracing research
**Reference-list screening**	Supplementary retrieval

S2.3 Search Limits

Parameter	Specification
**Search window**	January 2010 – December 2025
**Final search update**	January 2026
**Language**	English
**Publication types**	Empirical studies, dissertations, and proceedings papers
**Population**	Human participants in writing contexts

S2.4 Core Conceptual Search Structure

Cluster A: AI-Mediated Feedback	Cluster B: Process-Tracing Methodologies	Cluster C: Writing Contexts
• automated writing evaluation<br>• AWE<br>• AWCF<br>• AI feedback<br>• generative AI<br>• GPT<br>• ChatGPT	• “keystroke logging”<br>• “eye tracking”<br>• “screen capture”<br>• “interaction logs”<br>• “revision logs”<br>• “process tracing”<br>• “draft history”	• “L2 writing”<br>• ESL<br>• EFL<br>• “second language writing”<br>• revision<br>• “feedback uptake”

• large language model	• “timestamped analytics”	• “academic writing”

S2.5 Representative Database Search Strings

**Web of Science Core Collection**<br>`TS=("automated writing evaluation" OR AWE OR AWCF OR "AI feedback" OR "generative AI" OR GPT OR ChatGPT) AND("keystroke logging" OR "eye tracking" OR "process tracing" OR "revision logs")AND("L2 writing" OR ESL OR EFL OR "second language writing")`
**Scopus**<br>`TITLE-ABS-KE("AI feedback" OR "automated writing evaluation" OR ChatGPT (or GPT, or "large language model")AND("keystroke logging" OR "interaction logs" OR"process tracing")AND("L2 writing" OR EFL OR ESL)`
**ERIC**<br>`("AI feedback" OR "automated writing evaluation")AND("revision behavior" OR "process tracing")AND("second language writing" OR ESL OR EFL)`

S2.6 Supplementary Retrieval Procedures

To enhance retrieval completeness, supplementary screening procedures included:

- manual review of reference lists from eligible studies,
- targeted searches of relevant conference proceedings,
- and screening of related empirical literature identified during full-text review.

S2.7 Duplicate Removal and Screening Procedures

Retrieved records were screened systematically following duplicate removal and eligibility assessment procedures. Title–abstract screening and full-text evaluation were conducted independently by two reviewers in accordance with the review protocol.

Supplementary File S3. Data Extraction and Coding Framework

S3.1 Extraction Framework

A structured extraction framework was developed to standardize coding across heterogeneous study designs, tracing methodologies, and AI-feedback environments.

Extraction procedures focused on:

- participant characteristics,
- AI-system variables,
- process-tracing indicators,
- behavioral uptake patterns,

- methodological quality,
- and reported outcomes.

S3.2 Extraction Domains

Domain	Extracted Information
Study metadata	Author, year, publication source
Participant variables	Sample size, proficiency, educational level
Writing-task variables	Genre, timing, revision context
AI variables	Tool type, feedback function
Temporal indicators	Pauses, latency, revision duration
Cognitive indicators	Monitoring, evaluation, decision-making
Behavioral indicators	Uptake, rejection, recursive revision
Methodological indicators	Calibration, timestamp precision
Outcomes	Writing performance and regulation indicators

S3.3 Temporal Coding Categories

Code	Operational Focus
**T1**	Pause duration before revision
**T2**	Burst-writing sequences
**T3**	Feedback-response latency
**T4**	Revision duration

S3.4 Cognitive–Metacognitive Coding Categories

Code	Operational Focus
**C1**	Monitoring behaviors
**C2**	Evaluative comparison
**C3**	Problem detection
**C4**	Revision decision-making

S3.5 Behavioral Uptake Categories

Code	Description
**B1**	Full acceptance
**B2**	Partial modification
**B3**	Selective rejection
**B4**	Clarification requests
**B5**	Recursive revision cycles

S3.6 Reliability Procedures

A subset of included studies was independently double-coded to evaluate coding consistency and procedural stability. Inter-coder agreement was calculated using Cohen's κ across major coding categories. Discrepancies were resolved through discussion and iterative refinement of coding definitions.

Supplementary File S4. Risk-of-Bias and Methodological Quality Appraisal Framework

S4.1 Appraisal Procedures

Methodological quality appraisal was conducted using:

1. The Mixed Methods Appraisal Tool (MMAT, 2018),
2. supplemented by process-tracing-specific quality indicators relevant to AI-mediated writing research.

S4.2 MMAT Evaluation Domains

Domain	Evaluation Focus
Sampling adequacy	Participant selection and representativeness
Design appropriateness	Alignment between design and research aims
Data-collection integrity	Adequacy of trace collection
Analytic rigor	Transparency and coherence of analyses
Interpretive validity	Alignment between evidence and conclusions

S4.3 Process-Tracing Quality Indicators

Trace Precision

Evaluation of: timestamp granularity, event continuity, and synchronization integrity.

Instrument Calibration

Evaluation of: eye-tracker calibration reporting, keystroke logger validation, and trace-recording accuracy.

AI Transparency

Evaluation of: model disclosure, version reporting, and feedback-system specification.

Reproducibility

Evaluation of: transparency of analytic procedures, operational coding definitions, and reporting clarity.

Data Completeness

Evaluation of: missing trace disclosure, incomplete logging, and handling of unavailable process data.

S4.4 Risk-of-Bias Classification

Classification	Interpretation
**Low risk**	Strong methodological transparency and trace integrity
**Moderate risk**	Some reporting or methodological limitations
**High risk**	Major limitations affecting interpretability or reproducibility

S4.5 Appraisal Reliability

Risk-of-bias evaluations were conducted independently by two reviewers. Disagreements were resolved through structured discussion and consensus procedures.

S4.6 Sensitivity Analysis

Sensitivity analyses were conducted to examine the stability of synthesis patterns after excluding studies with higher methodological risk.
